# Rhabdomyolysis with compartment syndrome-induced acute kidney injury in resource-limited settings: A case report

**DOI:** 10.22088/cjim.13.4.810

**Published:** 2022

**Authors:** I Gede Yasa Asmara, Henry Pebruanto, I Made Arya Winatha

**Affiliations:** 1 Department of Internal Medicine, Faculty of Medicine, University of Mataram - West Nusa Tenggara General Hospital, Mataram, Indonesia; 2 Department of Orthopedic Surgery, Siloam Hospitals Mataram, Mataram, Indonesia; 3 Department of Surgery, Faculty of Medicine, University of Mataram - West Nusa Tenggara General Hospital, Mataram, Indonesia

**Keywords:** Acute kidney injury, Compartment syndrome, Rhabdomyolysis

## Abstract

**Background:**

Diagnosis and management of rhabdomyolysis-induced acute kidney injury (AKI) are challenging in resource-limited settings. Laboratory markers for the diagnosis of rhabdomyolysis and continuous renal replacement therapy (CRRT) for the management of unstable hemodynamic AKI may be difficult to access. This report presented a case of rhabdomyolysis with compartment syndrome, which had a high prognostic factor for kidney failure and death in Lombok, Indonesia.

**Case presentation:**

A 34-year-old man came to the hospital complaining of pain and swelling in his right thigh after being buried by an avalanche of buildings. Laboratory examination showed leukocytosis, hemoconcentration, increased creatinine, metabolic acidosis, hyperkalemia, and dark brown urine. Muscle damage markers showed levels of creatinine phosphokinase >20000 U/L, aspartate aminotransferase 255 U/L, alanine aminotransferase 186 U/L, and lactate dehydrogenase >3000 U/L. Diagnosis of rhabdomyolysis, compartment syndrome, and AKI was primarily on clinical grounds. Despite immediate management (fluid therapy, antibiotics, and fasciotomy), the patient continued progress to AKI. Because CRRT was not available, the patient received a single hemodialysis treatment. A day later, the patient developed hypotension, went into septic shock, and died after five days of treatment.

**Conclusion:**

A patient with rhabdomyolysis, compartment syndrome, and acute kidney injury could have a better outcome if the patient arrived early and is treated immediately in a fully-equipped health care facility.

Rhabdomyolysis is a condition resulting from the destruction of damaged muscles and the release of the intracellular components of the muscle (1). Trauma is the cause of 8.8-26.7% of all cases of rhabdomyolysis (2). In rare scenarios, extensive rhabdomyolysis can develop into compartment syndrome that worsens muscle injury and results in acute kidney injury (AKI) (3). Rhabdomyolysis is usually characterized by muscle pain, swelling, weakness, and dark urine (4). Myoglobin, creatinine phosphokinase (CPK), and lactate dehydrogenase (LDH) are important markers of muscle damage (5). Diagnosis of rhabdomyolysis is challenging in resource-limited settings since the laboratory markers are not readily available. Continuous renal replacement therapy (CRRT) for AKI patients with unstable hemodynamic is also only available in capital or big cities in many developing countries. Here, we reported a case of rhabdomyolysis with compartment syndrome, which had a high risk of AKI and fatal outcome in Lombok, Indonesia.

## Case Presentation

**Figure 1:**
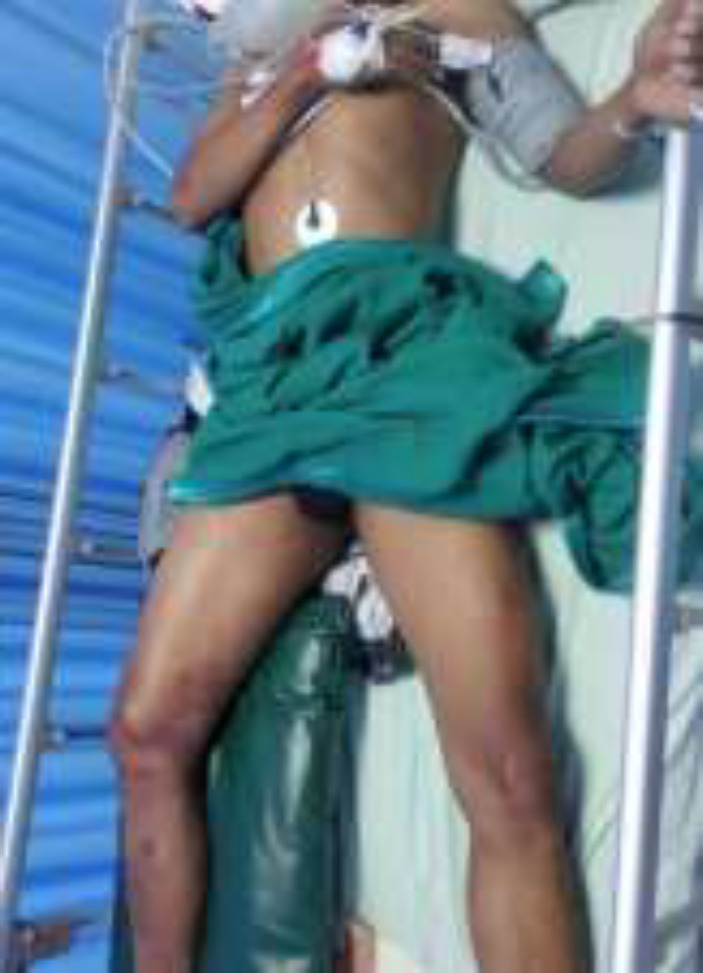
The right thigh of the patient looks bigger than the left thigh

A 34-year-old man came to the emergency department with complaints of pain and swelling in his right leg. He was a construction worker who had an accident buried by landslides while working 9 hours before admitted to the hospital. The avalanche hit his right leg at the level of his groin for about 5 hours, before he could be released by the evacuation team. A complete physical examination has been conducted on the following; body mass index 21.5 kg/m^2^, blood pressure 113/62 mmHg, pulse rate 112 x/min, respiration rate 26 x/minute, temperature 36.3 °C, and oxygen saturation 98%. While on local physical examination, the right thigh looked bigger than the left thigh (shown in figure 1). 

**Figure 2:**
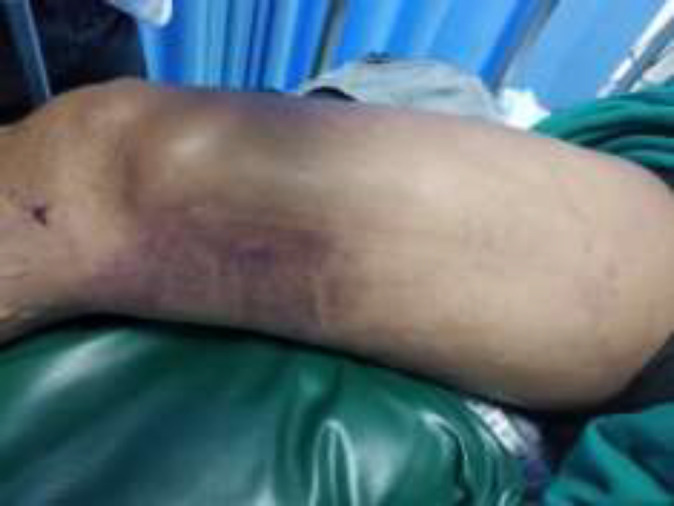
Edema, tension, and visible bruising on the right thigh of the patient

**Figure 3:**
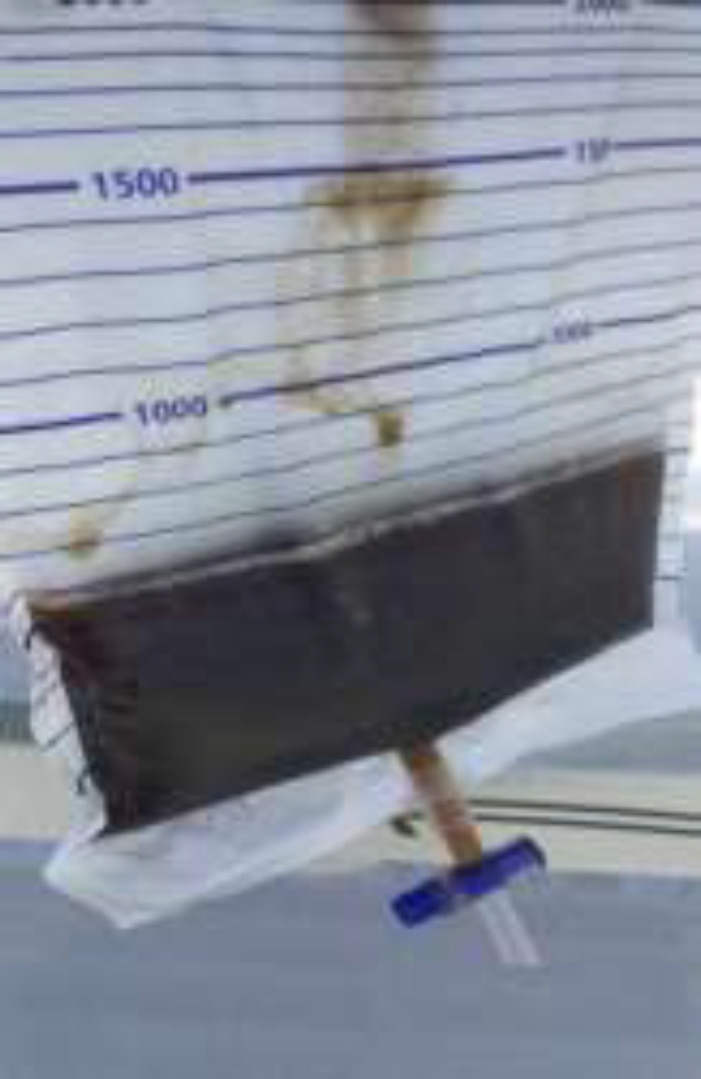
Dark brown-colored urine of the patient

Moreover, there was tenderness, tension, and visible bruising on the right thigh (shown in figure 2). The dorsalis pedis artery of the right leg was not palpable, and the right foot was cold and cyanosis. There were no signs of fracture in both legs. After insertion of a urinary catheter, dark brown urine was obtained (shown in figure 3). Doppler ultrasound examination of the right leg demonstrated a good blood flow to the femoral artery, but no flow to the popliteal artery, the posterior tibial artery, and the dorsalis pedis artery. Laboratory tests were performed and the results were the following; leukocytes 48.2 x10^3^/uL (neutrophils 91%), hemoglobin 21.4 g/dL, hematocrit 60.7%, platelets 344 x10^3^/uL, urea 46.1 mg/dL, creatinine 2.0 mg/dL, glucose 187 mg/dL, sodium 141 mmol/L, potassium 6.8 mmol/L, chloride 103 mmol/L. The results of blood gas analysis showed pH 7.15, PCO2 22.5, PO2 248, HCO3 7.8, and base excess -21. Macroscopic urine examination showed dark brown, cloudy, density 1.025, pH 6.0, negative leukocytes, blood 2+ (50), protein 1+ (30). Microscopic urine examination found 1-3 epithelium and 1-2 leukocytes. Markers of muscle damage showed levels of CPK >20000 U/L, aspartate aminotransferase (AST) 255 U/L, alanine aminotransferase (ALT) 186 U/L, and LDH >3000 U/L.

The initial diagnosis of the patient was rhabdomyolysis-induced AKI stage II. The initial treatment was 1 litter of ringers lactate infusion in 1 hour for 2 hours then maintained at 3000 cc/24 hours, 1 gram of calcium gluconate every 8 hours, 5 mg of salbutamol nebulizer every 8 hours, a mixture of 50 grams of dextrose plus 20 units of insulin every 8 hours, 40 mg furosemide every 8 hours, bolus 25 meq sodium bicarbonate and intravenous drip 100 meq in 12 hours, antibiotics ceftriaxone 1 gram every 12 hours, folic acid 400 mcg twice daily, and curcumin extract thrice daily. During short observation in the emergency unit, the patient was clinically suspected to have a complication of compartment syndrome. Afterwards, the patient underwent anterolateral fasciotomy for the right thigh compartment syndrome 6 hours after admission to the hospital. After surgery, the patient was transferred to the intensive care unit (ICU). 

During the observation, the blood pressure decreased and the patient developed oliguria. Administration of intravenous fluids was then limited to 1500 cc/24 hours, furosemide was stopped, and the vasopressor drug dobutamine 3 mcg/kg/minute was given. After 12 hours of treatment, complete blood count showed improvement, leukocytes 33.6 x10^3^/µL (neutrophils 93%), hemoglobin 16.3 g/dL, hematocrit 47.8%, platelets 195 x10^3^/µL. However, evaluation of renal function 24 hours after treatment showed worsening, namely urea 73.9 mg/dL, creatinine 3.2 mg/dL with blood gas analysis of pH 7.02, PCO2 43, PO2 214, HCO3 11.1, base excess -19.9, sodium 133 mmol/L, potassium 6.5 mmol/L. The patient was assessed for AKI stage III complicating with metabolic acidosis and hyperkalemia. 

Finally, the patient received 3 hours of hemodialysis (HD) treatment with temporary double-lumen access, blood velocity 150-200 cc/minute, dialysate velocity 400 cc/minute, ultrafiltration volume 0.9 litters, ultrafiltration coefficient 1.5, and minimal heparin anticoagulant. During the initial HD treatment, the patient's blood pressure dropped so the patient was given an additional 0.1 mcg/kg/min and up-titration of intravenous norepinephrine. As soon as the first HD was completed, the patient was transferred to a referral hospital in the capital city. Laboratory tests after the initial HD treatment revealed urea at 67.1 mg/dL, creatinine 3.4 mg/dL, sodium 146 mmol/L, potassium 4.3 mmol/L, chloride 99 mmol/L. At the referral hospital, the patient remained hemodynamically unstable. Subsequent HD treatment was postponed because the patient had low blood pressure while on vasoactive agents. The patient's condition continued to deteriorate and eventually died from AKI complications and possibly septic shock after 5 days of hospitalization. Written informed consent from the patient’s next of kin has been obtained for publication including images. This study was approved by the Ethics Committee for Medical Research of University of Mataram (115/UN18.F7/ETIK/2021).

## Discussion

AKI is the most serious complication of rhabdomyolysis that occurs in 17-65% of cases (1,6). A study reported that among the 341 patients with rhabdomyolysis, 51% developed AKI, and 20% required dialysis therapy (6). A history of alcohol consumption or drug use is a significant predictor of rhabdomyolysis (2,7), but there was no such history in this case. The main symptoms of rhabdomyolysis were muscle pain, muscle weakness, and muscle swelling (1). Compression and prolong immobilization due to trauma was the cause of rhabdomyolysis in this patient. The patient complained of pain, swelling, and weakness in the right leg developed AKI and received dialysis therapy.

Possible vascular involvement in this patient can be explained by the no flow of the popliteal, the tibialis posterior, and the dorsalis pedis arteries on doppler ultrasound examination. In general, muscle damage begins after 2 hours of ischemia and reaches a peak of 6-7 hours after ischemia (2). In general, AKI in rhabdomyolysis is caused by oxidative stress, inflammation, vasoconstriction, hypovolemia, tubular obstruction, tubular ischemia, and apoptosis (1,4). Rhabdomyolysis progressed to AKI because the patient developed oliguria, the creatinine increased, and the urine turned dark brown. The patient's urine examination showed a positive dipstick for blood but no erythrocytes in the urine.

Increased levels of CPK are sufficient to diagnose rhabdomyolysis, particularly where the increased level is five times the normal, approximately above 1000 IU/L (1,4,8,9). Correlation between CPK levels and AKI was stronger in trauma cases (10). Other laboratory tests might show an increased level of muscle enzymes such as LDH, AST, and ALT. Hyperuricemia, hyperkalemia, hyponatremia, metabolic acidosis, hypo or hypercalcemia, and hyperphosphatemia are sometimes found (1,4,5,11). Although kidney biopsy and myoglobin levels were not measured in this case, the diagnosis of rhabdomyolysis related to AKI can be established clinically (8). Myoglobin examination is not available in many places. Moreover, myoglobin has a very short half-life, high clearance ratio, and is excreted very quickly so it is difficult to detect (12). In this case, the levels of CK, LDH, AST, and ALT were elevated, accompanied by hyperkalemia and metabolic acidosis due to AKI. Rhabdomyolysis-induced AKI usually presents with a volume-depleted state due to fluid sequestration to the injured muscle (4). 

Inadequate fluid intake before the evacuation and rhabdomyolysis explained the condition of dehydration in the patient at admission. Fluid therapy should be done as early as possible, within the first 6 hours after muscle injury. Fluids should be given with a target of urine production of 300 cc/hour or more in the first 24 hours unless there are limiting conditions such as oliguria, fluid overload, and pulmonary edema (12). The patient was administered adequate fluid resuscitation but the presence of oliguria and possible volume expansion limit the intravenous fluid administration in this patient. The patient was given a broad-spectrum of antibiotics because the secondary bacterial infection cannot be excluded if the white blood cells are very high. Pain, palpable swelling, and absent peripheral pulses were 3 out of the 5 signs of compartment syndrome in this patient. The patient was diagnosed clinically since compartment pressure measurements were not available. 

Right thigh fasciotomy with an anterolateral approach was performed by the orthopedic surgeon six hours after admission. During ICU observation after surgery, the decreased blood pressure of the patient can result from the combination of metabolic acidosis and septic shock. Postoperative laboratory results revealed increased creatinine, decreased blood pH, and high levels of white blood cells with a predominance of neutrophils. HD therapy is only supportive and unable to eliminate myoglobin due to its large molecular weight. The aim of dialysis is only to improve impaired kidney function, overcome uremia, and fluid overload (8,9). When the patient's condition is unstable with hypotension and vasoconstrictor support, it requires CRRT. But CRRT was unavailable due to limited facilities and resources. CRRT was associated with a more stable hemodynamic patient and slightly eliminating myoglobin (1). The use of highly permeable membranes in continuous venovenous hemofiltration might be a promising therapy because of its ability to filter large molecules such as myoglobin (4). After finishing initial HD treatment, the patient's condition continued to deteriorate. The patient remained hypotension dependent on a combination of the vasopressor agent and eventually died because of septic shock. Many factors could contribute to the patient’s mortality, namely prolong patient evacuation, late arrival, late referral, and unavailability of CRRT.

The mortality rate of AKI due to rhabdomyolysis ranges from 3-46% (6). Based on the risk score developed by McMahon, which predicts the occurrence of kidney failure and death in rhabdomyolysis patients (13), the score for this patient was at least 8.5 (not yet including calcium and phosphate points). The risk is low if the score is less than 5 (mortality rate 2.3%) and high risk, if the score is more than 10 (mortality rate 61.2%) (1,13). The limitation of this case was the calcium and phosphate level unknown due to limited laboratory facilities. Therefore, the total predictive score of mortality in these patients cannot be calculated.

In conclusion, the patient came already in a condition of rhabdomyolysis, complicated into compartment syndrome, and ended in AKI. The patient had a high predictive risk of kidney failure and death. The management of fluid therapy and dialysis is only supportive and has limited ability to prevent the adverse effects of myoglobin on the kidneys. If the patient has arrived earlier and the management has been done immediately in a fully-equipped health care facility, the patient might have had a better outcome.

## Acknowledgments

The authors would like to thank the next of kin of deceased patient in the agreement for publication, and to Dr. Muhyiddin and Dr. Joanna for helping us in collecting the information.

### Funding:

No particular funding was obtained for this case report.

### Conflict of Interest: 

The authors have no conflict of interest to declare. 

### Informed consent:

Informed consent was obtained from his next of kin. 

### Author contributions:

 IGYA was responsible for the study concept and design; IGYA, HP, IMAW treated the patient during hospitalization; IGYA wrote the first draft of the manuscript; IGYA and IMAW revised the manuscript; All authors critically reviewed and approved the final manuscript.
